# YouTube as a source of information and education on endometriosis

**DOI:** 10.1097/MD.0000000000030639

**Published:** 2022-09-23

**Authors:** Kyong-No Lee, Hyun-Jin Tak, So-Yoon Park, Sung Taek Park, Sung-Ho Park

**Affiliations:** a Department of Obstetrics and Gynecology, Seoul National University Bundang Hospital, Seongnam, Korea; b Department of Obstetrics and Gynecology, Kangnam Sacred Heart Hospital, Hallym University Hospital, Seoul, Korea.

**Keywords:** endometriosis, internet, quality of information, YouTube

## Abstract

Many patients seek information online, including on social media, regarding various health topics. This study aimed to investigate whether YouTube videos on endometriosis could be a useful source for the general population, surgical trainees, and specialists. A YouTube search was conducted on December 26, 2021, using the search terms “endometriosis,” “endometrioma,” and “endometriotic cyst.” Videos were sorted by view count, and the 100 videos with the highest view counts were chosen. After excluding 48 videos for various reasons, 52 were included in the final analysis. The number of views, duration, likes and dislikes, content type, and source of each video were recorded. We referred to a previous study to evaluate video quality. The 52 videos related to endometriosis had a total of 35,220,141 views (median 233,688, range 48,874–10,452,366). Based on authorship, the videos were categorized into videos uploaded by the medical group and the nonmedical group. The medical group mainly uploaded videos directly related to endometriosis, such as explanations or detailed surgical procedures for endometriosis (26/27, 96%), whereas the nonmedical group mainly uploaded videos about personal experiences and others (24/25, 96%; *P <.*001). Evaluating the score by each type of content, videos containing personal experiences (median score 6, range 3–10) scored significantly lower than videos containing other content such as explanations of the disease (median score 14, range 7–18; *P < .*001) and surgical procedures (median score 9, range 5–17; *P* < .001). Analysis according to the source, the number of views and video power index was significantly higher in the videos uploaded by the nonmedical group *(P < .*05). YouTube is currently not an appropriate source for patients to gain information on endometriosis. Credible videos with accurate information and clear, high-quality operative clips with proper scientific commentary should be uploaded by medical professionals and medical institutions to critically and rapidly appraise the quality of online video-disseminated information on endometriosis. In addition, advanced filtering using categories by YouTube’s staff appears to be necessary.

## 1. Introduction

Endometriosis is a debilitating disease characterized by chronic inflammation and the presence of functional endometrial glands and stroma outside the uterine cavity.^[1]^ Endometriosis appears to be one of the most common benign gynecological proliferations in premenopausal women since it is estimated that 10%–15% of reproductive-age women suffer from pelvic endometriosis.^[2]^ The prevalence of this disease increases up to 30% in patients with infertility and up to 45% in patients with chronic pelvic pain.^[3,4]^ There are currently no diagnostic markers with adequate reliability for clinical use.^[5]^ Although a noninvasive diagnostic test for endometriosis is desirable and could help avoid the need for surgery in establishing a definitive diagnosis, no such test is available currently.^[6]^ Endometriosis is a chronic disease that requires sustained treatment; this key educational point must be reinforced in discussions with patients.^[7]^ As endometriosis has a common prevalence and its diagnosis is not easy, it is important to consult a gynecologist. However, when treatment is difficult, patients attempt to obtain information regarding the condition elsewhere.

The internet provides access to a wide range of online medical and visual educational resources.^[8]^ YouTube, one of the most common Internet-based visual information and entertainment platforms, boasts more than 2 billion video views every day.^[9]^ In addition, there is a high level of information posted by experts. However, because YouTube does not question the credibility of video creators, information that is inappropriate (or lacks expertise) is often posted. Although researchers representing various specialties have analyzed the accuracy of YouTube videos on various topics, controversy regarding the reliability of these videos due to the lack of peer review persists.^[10–14]^ There is one study on endometriosis and YouTube analyzing endometrioma surgery video,^[15]^ but there is no study on videos regarding general endometriosis. Because many endometriosis patients with increasing levels of anxiety,^[16]^ it is important to use reliable YouTube videos with appropriate information from video uploaders who do not lack expertise.

The primary purpose of this study was to analyze the content of the most viewed videos of endometriosis on YouTube to identify the features of endometriosis-related videos that were watched by the public. In addition, we evaluated the quality of videos related to endometriosis on YouTube to determine whether accurate and important information was delivered.

## 2. Materials and Methods

### 2.1. Search strategy

A YouTube search was conducted on December 26, 2021, using the terms “endometriosis,” “endometrioma,” and “endometriotic cyst.” The inclusion criteria for the videos were as follows: (1) English language used, (2) primary content related to endometriosis, and (3) acceptable audiovisual quality. The exclusion criteria were as follows: (1) languages other than English, (2) absence of audio or visual stimuli, and (3) duplicate videos.

**Figure 1.**
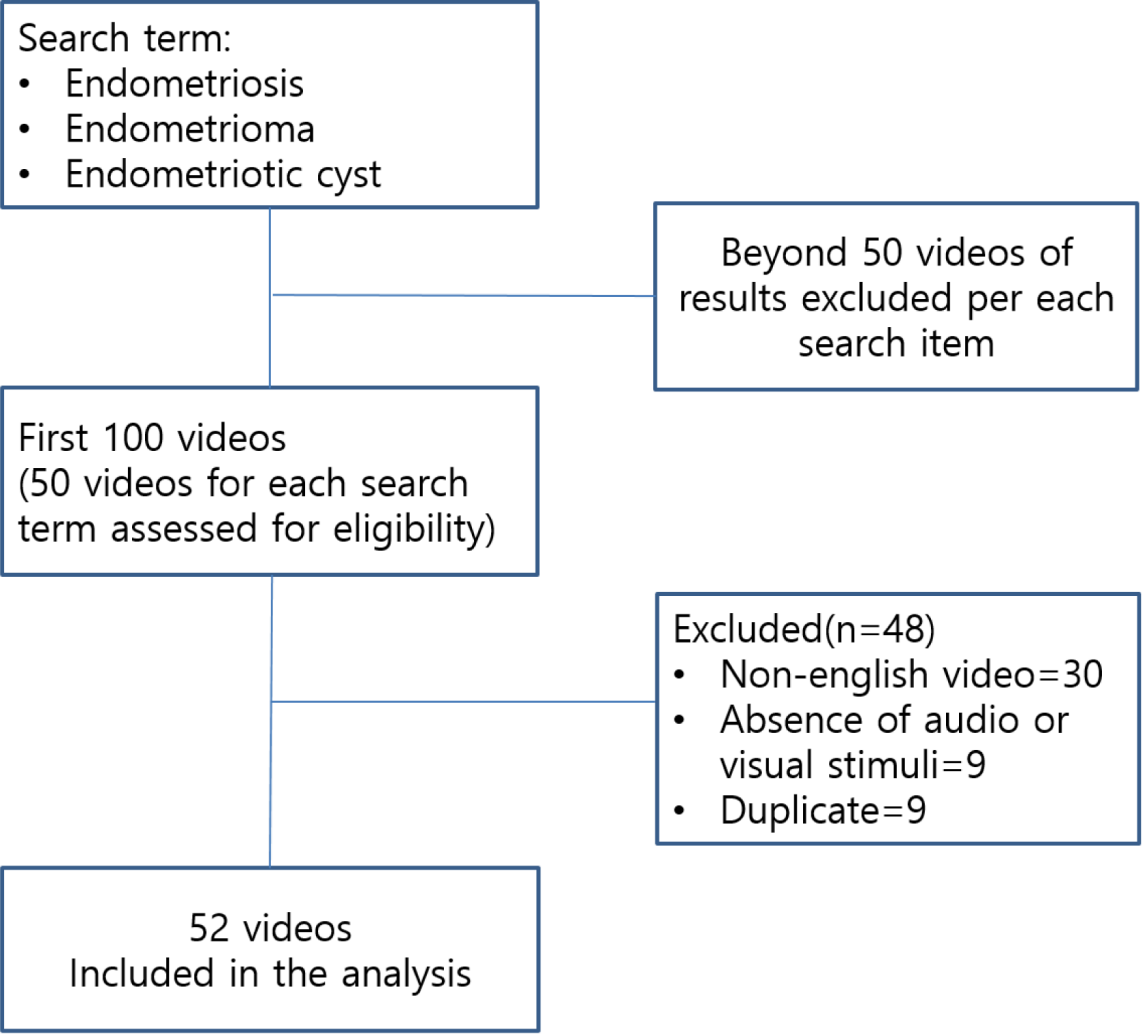
Methodology of selection of YouTube videos for the analysis.

A new account was created to avoid YouTube’s view of history-based video recommendations. The videos were sorted by the view count. For each search term, the top 50 of the 100 initial videos were included for review, as determined by the “relevance” filter, according to YouTube’s algorithm. A total of 48 videos were excluded (non English videos = 30, absence of audio or visual stimuli = 9, and duplicate videos = 9). Finally, 52 YouTube videos were found using the keywords “endometriosis,” “endometrioma,” and “endometriotic cyst” and were included in the final analysis (Fig. 1).

### 2.2. Video assessment

For each video, the following general parameters were noted: number of views, video length (min), total number of “likes” as depicted by the “thumbs up” icon, and purpose and type of content. Based on the contents of the video, they were categorized into 4 groups: explanations of the disease (providing medical information related to endometriosis, including diagnosis, symptoms, and treatment), surgical procedures (showing or explaining detailed surgical techniques and processes), personal experiences (sharing personal experiences and feelings related to endometriosis), and others (complementary treatment options available for endometriosis, including nutrition and exercise). Based on the source of upload, videos were classified into 5 basic groups: academics (authors were affiliated with a university), physicians (authors were not affiliated with a university but were physicians), patients (women who have been diagnosed with endometriosis and are currently undergoing treatment or have been treated), commercial establishments (attention to a product or service), and paramedical (allied health therapist, physiotherapist, or dietitian). We further categorized the videos uploaded by academic and physician groups into the medical group and those uploaded by patients, commercial establishments, and paramedical groups into the nonmedical group (Table 1).

**Table 1:** Characteristics of videos related to endometriosis on YouTube (n = 52).

Variables	Description	Value, n
Content		
Directly related		
Explanations of the disease	Provide medical information related to endometriosis, including medical treatment	15
Surgery videos	Show or explain detailed surgical procedures or techniques and processes	12
Indirectly related		
Personal experiences	Share personal experiences and feelings related to endometriosis	13
Others	Complementary treatment options available for endometriosis (e.g., nutrition and exercise)	12
Video authorship		
Medical		
Academic	Authors are affiliated with a university	13
Physician	Authors are not affiliated with a university but are physicians	14
Nonmedical		
Patient	Women who have been diagnosed with endometriosis and are currently undergoing treatment or have been treated	9
Commercial	Attention to a product or service	6
Paramedical	Allied health therapist, physiotherapist, or dietitian	10

Because there are no established standards for evaluating video quality, we prepared an arbitrary scoring system based on a previous study.^[17–20]^ The evaluation factors were divided into the part that evaluated the general quality of the video, whether important information on endometriosis was included and explained, and how much scientific evidence was specified. For general video quality and flow of video content, each parameter was scored on a scale of 1 to 3: poor, 1 point; moderate, 2 points; and good, 3 points. The information on endometriosis was divided into 5 elements (cause, symptoms, diagnosis, treatment, and recovery for a given health problem) and evaluated as follows depending on the degree of explanation: 0 points, not mentioned; 1 point, mentioned briefly; and 2 points, mentioned in detail. For videos based on scientific evidence, there were 2 subdivided items: 0 points were given if there was no mention and 1 point was given if there was any mention. Thus, the total score of the 5 items ranged from a minimum of 2 to a maximum of 18 points. Three physicians independently evaluated the quality of each video, and the average of the 3 scores was used for the 3 scores.

To assess the popularity of the videos, we used the like ratio (like×100/like), view ratio (number of views/day), and video power index (VPI) (like ratio × view ratio/100). In previous studies, dislike was included in the video evaluation index; however, due to YouTube’s recent policy, the number of dislikes was not indicated.

### 2.3. Statistical analysis

Data on video characteristics, including source, intent, and number of views since posting, were collected. Data were shown as median (range) for continuous variables and n (%) for categorical variables. Comparisons were made between the medical and nonmedical groups using the Mann–Whitney *U* test. Comparisons of the difference in uploaded contents between the medical and nonmedical groups were analyzed using Fisher’s exact test. Statistical analysis was conducted using the SPSS software (version 25.0, IBM, Armonk, NY, USA). A *P* value of < .05 was considered statistically significant. The reliability between YouTube videos and the 3 physicians scored on the criteria for the items was assessed using intraclass correlation coefficients (ICC).

### 2.4. Ethics statement

The need for institutional review board approval was waived for this study because only publicly available data were used.

## 3. Results

The 52 videos related to endometriosis had a total of 35,220,141 views (median 233,688, range 48,874–10,452,366). The descriptive features of endometriosis-related videos on YouTube are shown in Table 2. The median length was 6.80 min (range 0.20–43.20), and the majority of videos (39/52, 75%) did not exceed 12 min. Videos were uploaded to YouTube approximately 1521 days previously, on a median (range 14–5289). Although video searches were conducted through views, 2018 had the highest number of videos, followed by 2017 and 2019 (Table 3).

**Table 2:** Descriptive features of videos related to endometriosis on YouTube (n = 52).

	**Median (range**)
Views	233,688 (48,874–10,452,366)
Video length (min)	6.80 (0.20–43.20)
Time on YouTube (d)	1,521 (14–5,289)
Likes (thumbs-up)	2,300 (16–66,000)
View ratio	174.5 (13.8–128,057.9)
Video power index	3,450.0 (2.2–84,518,185.7)

**Table 3:** Number of videos included in the study by year of upload (n = 52).

Year	Uploaded videos, n
**2007**	1
**2008**	0
**2009**	0
**2010**	0
**2011**	2
**2012**	5
**2013**	2
**2014**	2
**2015**	2
**2016**	4
**2017**	8
**2018**	9
**2019**	8
**2020**	6
**2021**	3

Table 1 shows a description of the categorization according to video content and authorship. The most prevalent content (n = 15) was videos that contained an explanation of endometriosis, which provided medical information related to endometriosis, including diagnosis, symptoms, and treatment. In many cases (11/15, 73%), the videos were uploaded by academics. The second most commonly uploaded videos were those that shared personal experiences and feelings related to endometriosis. Twenty-seven and 25 videos were uploaded by the medical and nonmedical groups, respectively. When analyzing the relationship between the source of the videos and the content, the medical group mainly uploaded videos directly related to endometriosis, such as explanations or detailed surgical procedures for endometriosis (26/27, 96%), whereas the non-medical group mainly uploaded videos about personal experiences and others (24/25, 96%; *P < .*001). To evaluate whether the videos related to endometriosis contained accurate and important information and whether scientific evidence was presented, we created a detailed scoring method. The median score was 8 (range 2–18); the video with the highest score was a video containing a well-organized general explanation of endometriosis, and the video with the lowest score was mainly composed of personal experience without including contents related to endometriosis. When evaluating the score by each type of content, videos containing personal experience (median score 6, range 3–10) scored significantly lower than videos containing other content, such as explanations of the disease (median score 14, range 7–18; *P < .*001) and surgical procedures (median score 9, range 5–17; *P* < .001). There was a high degree of correlation between reviewers (ICC 0.956, 95% CI, 0.930–0.973; *P < .*001). Then, we created and analyzed the like ratio, view ratio, and VPI to evaluate which videos people were interested in and liked. The video with the highest VPI was a short video posted by Beeston Fam, which was a short video of only 12 s. In addition, the title of this video is when the only treatment for endometriosis is to get pregnant over and over #shorts, but this video had nothing to do with endometriosis. We further analyzed how videos related to endometriosis uploaded on YouTube differed according to the uploaded content, source, and time when the video was uploaded to YouTube. We compared videos uploaded by the end of 2018 and videos uploaded after 2018, which was time to compare by dividing the number of uploaded videos in half. We also compared videos based on the source. When analyzed according to the source (Table 4), the median number of views and degree of popularity of the videos represented by VPI and likes (thumbs-up) were significantly higher in the videos uploaded by the non-medical group *(P < .*05*).* However, videos uploaded by the medical group showed significantly higher scores than those by the nonmedical group (median 13, range 5–18, vs. median 6, range 2–10; *P < .*001). When analyzed according to the date when the video was uploaded (Table 5), likes (thumbs-up), view ratio, and VPI were significantly higher in the group with videos uploaded after 2018. However, there was no significant difference in the number of videos uploaded by the medical group and their scores.

**Table 4:** Comparison according to the source of the videos.

Variable	Value, median (range)		*P* value
	Medical group (n = 27)	Nonmedical group (n = 25)	
Views	148,735 (48,874–10,452,366)	246,630 (49,861–1,792,810)	.122
Video length (min)	6.85 (2.07–22.80)	6.73 (0.20–43.20)	.949
Time on YouTube (days)	1,744 (110–5,289)	1,361 (14–3,447)	.230
Likes (thumbs-up)	859 (16–36,000)	4,300 (514–66,000)	**.005**
View ratio	108.8 (13.8–7635.0)	185.8 (28.9–128,057.9)	.230
Video power index	907.9 (2.2–839,854.1)	9,770.6 (232.1–84,518,185.7)	**.030**
Score	13 (5–18)	6 (2–10)	**<.001**

**Table 5:** Comparison according to the time of upload of the videos to YouTube.

Variable	Value, median (range)		*P* value
	By the end of 2018 (n = 26)	After 2018 (n = 26)	
Views	164,500 (48,874–6,563,339)	272,431 (55,687–10,452,366)	.055
Video length (min)	5.37 (1.97–43.20)	7.90 (0.20–30.13)	.577
Likes (thumbs-up)	844 (16–36,000)	9,300 (22–66,000)	**.001**
View ratio	64.8 (13.8–2239.3)	393.7 (51.6–128,057.9)	**<.001**
Video power index	650.3 (2.2–806,141.9)	27619.3 (198.7–84,518,185.7)	**<.001**
Medical group video	61.5% (16/26)	42.3% (11/26)	.267
Score	6 (2–18)	9 (2–18)	.139

## 4. Discussion

We identified that the most viewed video about endometriosis was uploaded in 2018 by Drugs.com, which is a website that provides information on prescription drugs. This is a well-organized video containing information on the cause of endometriosis. The second most viewed video was uploaded in 2013 by Nucleus Medical Media, which is the same company that uploaded the video with the highest number of views and VPI even during a YouTube study related to cesarean section.^[20]^ These 2 videos showed the highest VPI values among the 52 videos, except for 1 video. The video with the highest VPI was uploaded in 2021 by the Beeston Fam; an individual belonging to the nonmedical group, and it was difficult to find a correlation with endometriosis in the video. Reflecting these results, VPI, which is a comprehensive indicator of popularity, was significantly higher in the non-medical group than in the medical group. However, video scores were significantly higher in the medical than in the nonmedical group. This shows that VPI does not reflect the quality and reliability of the information provided by YouTube videos and that laypeople expressed their preferences regardless of the quality of the video. These results are similar to those of previous studies. Kunze et al reviewed 50 videos regarding menisci and found that information on meniscus found in YouTube videos was of low quality and reliability.^[21]^ Ferhatoglu et al recently reported an association between high VPI scores and low DISCERN scores in their review of cardiopulmonary resuscitation videos on YouTube.^[22]^

There was no significant difference in the scores of the groups or videos uploaded before and after 2018, whereas the number of likes, view ratio, and VPI, which are indicators of the popularity of videos, were significantly higher in the group of videos uploaded after 2018. Considering that there is no difference in scores between the 2 groups and the increase in the number of video uploads after 2018, this can be interpreted as YouTube becoming more popular and laypeople can easily access and produce content.

**Figure 2.**
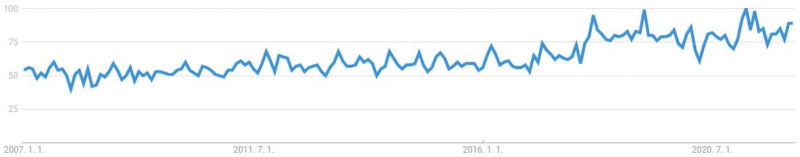
Search trend for the term *endometriosis* on YouTube. The search volume of endometriosis using YouTube is gradually increasing.

**Figure 3.**
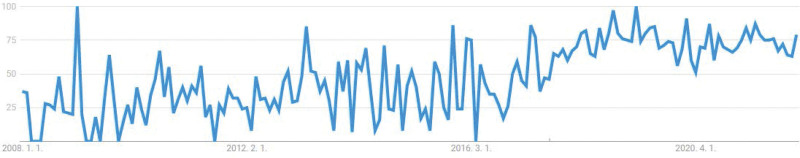
Search trend for the term endometriosis on Google website. The number of YouTube users and searches for “endometriosis” on the Google website has been increasing.

It can be seen that the search volume of endometriosis using websites or YouTube is gradually increasing (Figs. 2,3). In the case of YouTube, information can be transmitted without filtering or scientific verification. Therefore, we need higher quality videos, and YouTube must implement an active filtering process. We evaluate its usefulness to some extent by comparing the information available on YouTube with information from other sources. In addition, several studies have pointed out that YouTube still lacks the function of delivering medical information.^[13,23–29]^ However, recent studies on the benefits of YouTube are also increasing.^[30–32]^ Therefore, in the future, providing high-quality medical information using YouTube in the field of obstetrics and gynecology is a possibility.

This study has some limitations. We analyzed only 52 YouTube videos identified using the keywords “endometriosis,” “endometrioma,” and “endometriotic cyst.” It was classified according to the number of views, and only videos in English were included; thus, a sampling bias may have occurred. The scoring system was based on previous studies; however, there is still no unified standard for evaluating videos. Thus, more verification is needed to ensure that the assessment method is suitable for accurately evaluating the quality of endometriosis videos.

Our study found that YouTube videos regarding endometriosis were misleading or inaccurate and presented a risk of harmful consequences. The results suggest that the quality of information in videos on endometriosis is poor, and YouTube is currently not an appropriate source of such information for patients with endometriosis. Credible videos with accurate information and clear, high-quality operative clips with proper scientific commentary should be uploaded by medical professionals and medical institutions to critically and rapidly appraise the quality of online video-disseminated information on endometriosis. In addition, advanced filtering using categories by YouTube’s staff appears to be necessary.

## Author contributions

Conceptualization: Kyong-No Lee, Sung-Ho Park.

Data curation: Kyong-No Lee, Hyun-Jin Tak, So-Yoon Park.

Formal analysis: Kyong-No Lee, Hyun-Jin Tak, So-Yoon Park.

Funding acquisition: Sung Taek Park, Sung-Ho Park.

Investigation: Hyun-Jin Tak, So-Yoon Park, Sung Taek Park.

Methodology: Sung-Ho Park.

Project administration: Kyong-No Lee, Sung-Ho Park.

Resources: Hyun-Jin Tak, So-Yoon Park, Sung Taek Park.

Supervision: Sung Taek Park, Sung-Ho Park.

Validation: Sung Taek Park, Sung-Ho Park.

Visualization: Hyun-Jin Tak, So-Yoon Park.

Writing – original draft: Kyong-No Lee, Sung-Ho Park.

Writing – review & editing: Kyong-No Lee, Hyun-Jin Tak, So-Yoon Park, Sung Taek Park, Sung-Ho Park.
